# The impact of *HLA-G, LILRB1* and *LILRB2* gene polymorphisms on susceptibility to and severity of endometriosis

**DOI:** 10.1007/s00438-017-1404-3

**Published:** 2017-12-12

**Authors:** Aleksandra Bylińska, Karolina Wilczyńska, Jacek Malejczyk, Łukasz Milewski, Marta Wagner, Monika Jasek, Wanda Niepiekło-Miniewska, Andrzej Wiśniewski, Rafał Płoski, Ewa Barcz, Piotr Roszkowski, Paweł Kamiński, Andrzej Malinowski, Jacek R. Wilczyński, Paweł Radwan, Michał Radwan, Piotr Kuśnierczyk, Izabela Nowak

**Affiliations:** 1 Department of Clinical Immunology, Laboratory of Immunogenetics and Tissue Immunology, Hirszfeld Institute of Immunology and Experimental Therapy, Polish Academy of Sciences, ul. Rudolfa Weigla 12, 53-114 Wrocław, Poland; 2 Department of Histology and Embryology, Centre of Biostructure Research, Medical University of Warsaw, ul. Chałubińskiego 5, 02-004 Warszawa, Poland; 3 Department of Medical Genetics, Centre of Biostructure Research, Medical University of Warsaw, ul. Pawińskiego 3c, 02-106 Warszawa, Poland; 4 First Chair and Clinic of Obstetrics and Gynecology, Medical University of Warsaw, Pl. Starynkiewcza 1/3, 02-015 Warszawa, Poland; 5 Second Clinic of Obstetrics and Gynecology, Medical University of Warsaw, ul. Karowa 2, 00-315 Warszawa, Poland; 6 Department of Gynecology and Gynecological Oncology, Military Medical Institute, Central Clinical Hospital of Ministry of Defence, ul. Szaserów 128, 04-141 Warszawa, Poland; 7 Department of Surgical, Endoscopic and Oncologic Gynecology, Polish Mothers’ Memorial Hospital–Research Institute, ul. Rzgowska 281/289, 93-338 Łódź, Poland; 8 Department of Surgical and Oncological Gynecology, Medical University of Lodz, Al. Kościuszki 4, 90-419 Łódź, Poland; 9 Department of Reproductive Medicine, Gameta Hospital, ul. Rudzka 34/36, 95-030 Rzgów, Poland; 10 Biogeno – Regional Science-Technology Centre, Podzamcze 45, 26-060 Chęciny Kielce, Podzamcze, Poland; 11 Faculty of Health Sciences, The State University of Applied Sciences in Plock, Plac Dąbrowskiego 2, 09-402 Płock, Poland

**Keywords:** Endometriosis, KIR2DL4, LILRB, HLA-G

## Abstract

Endometriosis is a disease in which endometriotic tissue occurs outside the uterus. Its pathogenesis is still unknown. The most widespread hypothesis claims that ectopic endometrium appears as a result of retrograde menstruation and its insufficient elimination by immunocytes. Some reports have shown expression of non-classical HLA-G molecules on ectopic endometrium. HLA-G is recognized by KIR2DL4, LILRB1 and LILRB2 receptors on natural killer (NK) and other cells. These receptors are polymorphic, which may affect their activity. In this study we investigated whether *HLA-G, KIR2DL4, LILRB1* and *LILRB2* polymorphisms may influence susceptibility to endometriosis and disease progression. We used polymerase chain reaction (PCR), PCR-restriction fragment length polymorphism (PCR-RFLP) and allelic discrimination methods with TaqMan SNP Genotyping Assays for typing of 276 patients with endometriosis and 314 healthy fertile women. The *HLA-G *rs1632947:GG genotype was associated with protection against the disease and its severe stages; *HLA-G* rs1233334:CT protected against progression; *LILRB1* rs41308748:AA and *LILRB2* rs383369:AG predisposed to the disease and its progression. No effect of *KIR2DL4* polymorphism was observed. These results support the role of polymorphisms of HLA-G and its receptors LILRB1 and LILRB2 in susceptibility to endometriosis and its progression.

**Electronic supplementary material:**

The online version of this article (10.1007/s00438-017-1404-3) contains supplementary material, which is available to authorized users.

## Introduction

Endometriosis is an estrogen-dependent gynecological disease, affecting about 10% of women in reproductive age. It is associated with the occurrence of endometrium outside the uterus. Endometriotic lesions can be found mainly in the ovaries and pelvic peritoneum, but also in the rectovaginal septum, and at more distant locations such as the lung, liver, and pancreas, and even in scars after operative surgery (Ahn et al. 2015; Serdar and Bulun 2009; Gupta et al. 2016; Parkin and Fazleabas 2016; Vercellini et al. 2014). In addition, endometriotic lesions may undergo malignant transformation (Worley et al. 2013). The etiopathology of endometriosis is still poorly understood. One hypothesis of endometriosis development is Sampson’s theory of retrograde menstruation (Sampson 1927; Dastur et al. 2010). According to this theory, retrograde menstruation may result in implantation, survival and growth of endometrial cell foci in the peritoneal cavity. The mechanism(s) of this phenomenon is unknown; it is plausible, however, that it may be due to insufficient elimination of endometrial cells by the local immune system. Indeed, women with endometriosis were found to have reduced activity of natural killer (NK) cells (Oosterlynck et al. 1992; Maeda et al. 2012; Eidukaite and Tamosiunas 2008; Tariverdian et al. 2009). These cells, granular cytotoxic lymphocytes, have been found not only in the peripheral blood, but also in the peritoneal fluid (Eidukaite and Tamosiunas 2008; Králíčková and Vetvicka 2015; Kawashima et al. 2009). A defect of the NK activity in the recognition and lysis of implanted endometrial cells may be thus one of the crucial mechanisms in the initiation and progression of endometriosis. NK cell activity is regulated by different receptors—with activating or inhibitory action—such as killer immunoglobulin-like receptors (KIRs) and leukocyte immunoglobulin-like receptors (LILRs) (Maeda et al. 2012; Králíčková and Vetvicka 2015; Borges et al. 1997; van der Touw et al. 2017). KIR and LILR recognize class I human leukocyte antigens (HLA), among them HLA-G. HLA-G is expressed by placental trophoblasts, and it is known as a crucial factor in maintaining pregnancy. However, it may also be expressed on ectopic endometrial tissue in the peritoneal cavity and be recognized by immune cells via its receptors: KIR2DL4 (of both inhibitory and activating potential), and inhibitory LILRB1 and LILRB2 (Maeda et al. 2012; Kawashima et al. 2009; Wang et al. 2008; Hudson and Allen 2016; Kang et al. 2016). Moreover, HLA-G up-regulates LILRB1, LILRB2 and KIR2DL4 expression in antigen-presenting cells, NK cells, and T cells (LeMaoult et al. 2005).

Previous GWA studies of endometriosis have implicated *WNT* (wingless-type MMTV integration site) signaling and oestrogen responsive genes, genes involved in the actin cytoskeleton and cellular adhesion (Rahmioglu et al. 2014; Nyholt et al. 2012), the *CDKN2BAS* locus encoding the cyclin-dependent kinase inhibitor 2B antisense RNA (Uno et al. 2010), and four single nucleotide polymorphisms (SNPs) located in and around interleukin 1α (Adachi et al. 2010). Most of the identified GWAS variants were non-coding. The most recently published studies by Sapkota et al. (2017a, b) have evaluated the potential role of coding variants in endometriosis risk by large exome-array analysis. However, their results did not identify any coding variants with MAF > 0.01, with moderate or large effect sizes in endometriosis pathogenesis. They provide genome-wide significant evidence for association with a splice variant (rs13394619) in the *GREB1* (Growth Regulation By Estrogen In Breast Cancer 1) locus in women with European ancestry. Moreover, the 19 SNPs identified in endometriosis explain up to 5.19% of variance in endometriosis, suggesting that many more variants remain to be detected. On the other hand, we focused rather on genes important for innate immune response. In our previous paper we found an association of NK cell receptor *KIR2DS5* gene and its potential ligand HLA-C C2 with endometriosis (Nowak et al. 2015a). Here, we analyzed other genes which may be involved in immune control of extra-uteral endometrial tissue. We examined the SNPs which may be associated with gene expression or splicing and therefore they could have potential influence on the receptor-ligand interaction between immune cells and ectopic endometrium.

Therefore, the aim of this retrospective study was to evaluate the association of the SNPs in genes coding for KIR2DL4, LILRB1 and LILRB2 receptors and their ligand HLA-G with susceptibility to and severity of endometriosis as potential non-invasive markers for the diagnosis of this disease.

## Materials and methods

### Study groups

The present study included 590 women from the Polish population who were enrolled during the period from 2005 to 2016. The study was approved by the Local Bioethics Committees at the Medical University of Wroclaw, Polish Mothers’ Memorial Hospital–Research Institute in Łódź, and the Medical University of Warsaw, Poland. Informed consent was obtained from all individual participants included in the study.

Endometriosis was diagnosed in 276 women. The patients were recruited at several Polish clinics: the First and Second Department of Obstetrics and Gynecology, Medical University of Warsaw; the Department of Surgical, Endoscopic and Oncologic Gynecology and the Department of Gynecology and Gynecologic Oncology in Polish Mothers’ Memorial Hospital–Research Institute in Łódź; and Gameta Hospital in Rzgów. The mean age of affected women was 33.02 ± 7.03 years. The diagnosis was based on laparoscopic surgery and confirmed by histopathological examination.

**Fig. 1:**
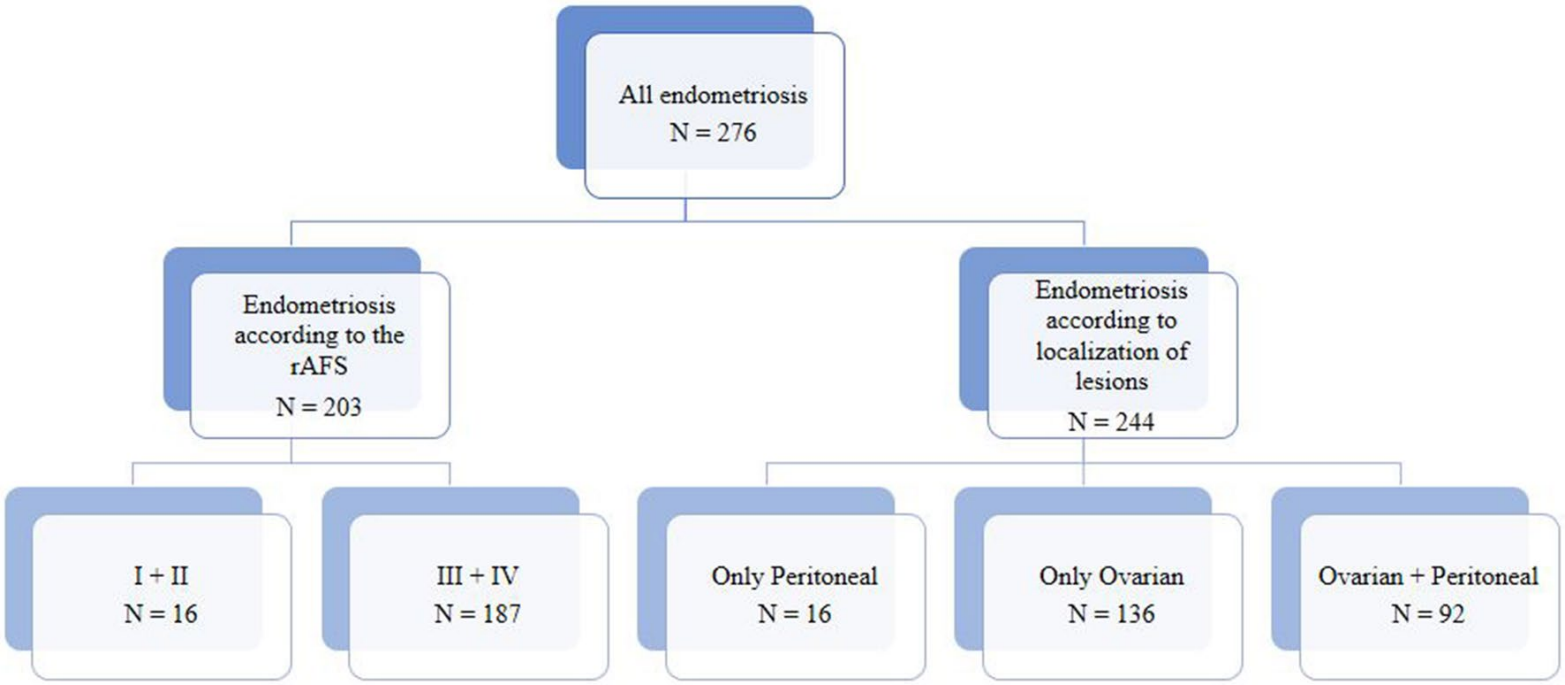
Flowchart of the study population

The patients were classified and analyzed according to the stage of the disease (American Fertility Society 1985) or according to the localization of the endometriotic lesions (Fig. 1). For 22 patients with endometriosis, detailed information on rAFS stage and lesion localization were not available.

The control group consisted of 314 fertile women. Among them 219 had at least two healthy-born children with the same partner without a history of spontaneous miscarriage and immunological or endocrinological diseases. Ninety-five women had at least one child. The mean age of fertile patients was 32.29 ± 5.81 years. The control group was recruited in the First Chair and Clinic of Obstetrics and Gynecology and the Department of Medical Genetics, University of Warsaw.

### DNA preparation and genotyping

Genomic DNA was isolated from 5 mL of the peripheral blood samples collected during the patient’s admittance to the hospital using the Invisorb Spin Blood Midi Kit (Invitek, Berlin, Germany) according to the producer’s instructions.

*HLA-G* genotyping was conducted in three sequence positions. To detect the 14 base pair insertion/deletion (rs371194629:c.*65_*66insATTTGTTCATGCCT) in the 3′ untranslated region (UTR) we used the polymerase chain reaction with sequence-specific primers (PCR-SSP) method. The rs1632947:A>G polymorphism was distinguished by real-time PCR. Details of the genotyping of these two polymorphisms have been previously described by Wiśniewski et al. (2010, 2015). The genotyping of the triallelic rs1233334:G>C/T was performed on a 7300 Real-Time PCR System (Applied Biosystems) using Applied Biosystems (Foster City, CA) ready-made Assay-on-Demand including two primers—forward 5′-ACTGTCTGGGAAAGTGAAACTTAAGAG-3′ and reverse 5′-AATGTGACTTTGGCCTGTTGGTATA-3′—and two fluorescently labeled probes: 5′-VIC-CTTTGTGAGTCGTGTTGTA-NFQ-3′ and 5′-FAM-CTTTGTGAGTCCTGTTGTA-NFQ-3′. The 10-μl reaction mixture contained ~ 20 ng of genomic DNA, 1 × TaqMan Universal PCR Master Mix, No AmpErase Uracil N-Glycosylase (UNG) (Applied Biosystems), primers and probes. PCR conditions were as follows: 95 °C for 10 min and (95 °C for 15 s, 60 °C for 1 min) × 40. This genotyping was confirmed by direct sequencing. Fig S1 shows the distribution of representative results in the scatter plot from the real-time PCR of *HLA-G* rs1233334:G>C/T SNP genotyping.

There are two variants of *KIR2DL4* with 9 or 10 consecutive adenines in the gene sequence. The deletion of one adenine in exon 7 contributes to the frame shift; therefore the 9A allele encodes the soluble form of the receptor with a missing transmembrane domain or truncated cytoplasmic tail. The 10A allele determines the membrane-bound receptor (Nowak et al. 2015b; Goodridge et al. 2007, 2009). The 10A/9A insertion/deletion in the 9620 position (rs11410751) of the *KIR2DL4* gene has been previously found in complete linkage disequilibrium with the rs649216:T>C of the gene (*r* ^2^ = 1) in our population (Nowak et al. 2015b). The T allele of the rs649216 corresponded to the 9A allele of the rs11410751, while rs649216:C corresponded to the variant with the 10A allele. Therefore, we decided to use the PCR method and restriction fragment length polymorphism (RFLP) with EarI digestion for testing of the rs649216:T>C *KIR2DL4* polymorphism, instead of the high resolution melting (HRM) method, which we found more expensive and troublesome than PCR-RFLP. Detailed protocols about these methods were published previously (Nowak et al. 2015b).

Genotyping of the rs41308748:G>A polymorphism in the *LILRB1* gene as well as the rs383369:G>A polymorphism and the rs7247538:C>T polymorphism in the *LILRB2* gene was carried out using PCR-RFLP. The restriction enzymes used in this study were as follows: AciI, TaiI and Hpy166II, respectively. The rs1061680:T>C in *LILRB1* was genotyped using the allelic discrimination method with TaqMan SNP Genotyping Assay (C_9491145_10) on a 7300 Real-Time PCR System (Applied Biosystems). Primer sequences, annealing temperatures, restriction enzymes and reaction conditions for *LILRB1* and *LILRB2* genotyping are listed in Table S1. Reference samples for all tested SNPs were sequenced by an external company (Genomed, Poland). Detailed information of all tested polymorphisms and their potential functions is summarized in Table 1.

**Table 1:** Summary of the tested SNPs

Chr	Gene	dbSNP ID	Position (bp)	Accession number	Reference sequence	Functional region	Potential effect
6	*HLA-G*	rs1632947	29,826,881	NC_000006.12	XM_017010817.1:c-964G>A	Promoter	Gene expression (Castelli et al. 2014)
6	*HLA-G*	rs1233334	29,827,120	NC_000006.12	XM_005249055.1:c.-725G>C XM_005249055.1:c.-725G>T	Promoter	Gene expression (Hviid et al. 2004, 2006)
6	*HLA-G*	rs371194629	29,830,804–29,830,805	NC_000006.12	NM_002127.5:c.*65_*66insATTTGTTCATGCCT	3′UTR of exon 8	mRNA stability; splicing; microRNA targeting (Castelli et al. 2014); the14 bp insertion allele is associated with lower concentration of soluble HLA-G (Chen et al. 2008)
19	*LILRB1*	rs41308748	54,636,725	NC_000019.10	NM_006669.6:c.1807-7G>A	Intronic	Splicing
19	*LILRB1*	rs1061680	54,632,001	NC_000019.10	NM_006669.6:c.425T>C	Non-synonymous, extracellular D2 domain, NP_006660.4:p. 142 Ile142Thr	Receptor-ligand interaction (Davidson et al. 2010; Kuroki et al. 2005)
19	*LILRB2*	rs7247538	54,278,869	NC_000019.10	NM_001080978.3:c.898C>T	Non-synonymous,NP_001074447.2:p. 300 His300Tyr	Splicing
19	*LILRB2*	rs383369	54,280,275	NC_000019.10	NM_001080978.3:c.59G>A	Signal peptide NP_001074447.2:p. 20 Arg20His	Gene expression (Hirayasu et al. 2008)
19	*KIR2DL4*	rs649216	54,813,180	NC_000019.10	NM_001080772.1:c.762T>C	NP_001074241.1:p. 254 Phe254	In complete LD with rs11410751(NC_000019.10:g.54813228_54813229insA), which determines the encoding of soluble or membrane-bound KIR2DL4 receptor (Nowak et al. 2015b)

*Chr* Chromosome; Genomic position is shown relative to GRCh38.p7; SNP IDs are according to dbSNP (rs, http://www.ncbi.nlm.nih.gov/SNP); c.*65_*66insATTTGTTCATGCCT was earlier described as 14 bp ins/del in 3′UTR of the *HLA-G* gene (Wiśniewski et al. 2010, 2015); NM_006669.6:c.1807-7G>A was earlier described as 5651 G>A (rs41308748) (Wiśniewski et al. 2015; Nowak et al. 2016); NM_006669.6:c.425T>C was earlier described as 927 T>C (rs1061680) (Davidson et al. 2010), and were relative to the translation start site

### Statistical analysis

SNP frequencies were estimated by direct counting. The statistical significance of differences in genotype and allele frequencies between the control group and patients was estimated using the two-sided Fisher’s exact test and by the Chi-square test with the appropriate degrees of freedom, *χ* ^2^ *df* (*df*=(*m* − 1) × (*n* − 1), where *m* = number of rows, *n* = number of columns). A *p* value of less than 0.05 was required to reject the null hypothesis, which assumes that there is no difference in the distribution of genotypes and alleles between the control group and patients. If *P* < 0.05, it was corrected (*P*_corr._) by the number of comparisons using Bonferroni correction. For 2 × 2 tables the odds ratio (OR) and 95% confidence interval for it were also calculated. Statistical analysis was performed using the software package GraphPad InStat version 3.06 (San Diego, CA, USA). Hardy–Weinberg equilibrium was checked using the Chi-square test with one degree of freedom for each SNP.

## Results

### *HLA-G* polymorphisms are associated with endometriosis

We found lower representation of rs1632947:GG genotype in patients with endometriosis than in controls (*P* = 0.04, *P*_corr__._ = 0.12, OR = 0.61, 95% CI = 0.39–0.96; Table 2). Limitation of our analysis to patients with known localization of lesions gave similar results but remaining even after correction (*P* = 0.009, *P*_corr._ = 0.027, OR = 0.53, 95% CI = 0.33–0.85; Table S2).

**Table 2:** *HLA-G* genotype and minor allele frequencies in women from Control and Endometriosis groups

Genotype	Control (%)	Patients (%)	Patients vs control				
			*P*	OR	95% CI	Test for independence	
						*p*	χ2
rs371194629:ins/del	*N* = 314	*N* = 276					
Del/del*	113 (35.99)	97 (35.14)		1		0.56	1.15
Ins/del	149 (47.45)	124 (44.93)	0.93	0.97	(0.68–1.39)		
Ins/ins	52 (16.56)	55 (19.93)	0.41	1.23	(0.77–1.96)		
Minor allele ins	253 (40.29)	234 (42.39)					
H-W	0.81	0.18					
rs1632947:G>A	*N* = 314	*N* = 276				0.08	4.97
AA*	63 (20.06)	76 (27.54)		1			
AG	157 (50.00)	131 (47.46)	0.08	0.69	(0.46–1.04)		
GG	94 (29.94)	69 (25.00)	**0.04** ^**a**^	**0.61**	**(0.39–0.96)**		
Minor allele A	283 (45.06)	283 (51.27)					
H-W	0.86	0.41					
rs1233334:G>C/T	*N* = 314	*N* = 276				0.65	2.48
CC*	215 (68.47)	188 (68.12)		1			
CG	79 (25.16)	70 (25.36)	1.00	1.01	(0.70–1.47)		
GG	8 (2.55)	6 (2.17)	1.00	0.86	(0.29–2.52)		
GT	1 (0.32)	4 (1.45)	0.19	4.57	(0.51–41.31)		
CT	11 (3.50)	8 (2.90)	0.82	0.83	(0.33–2.11)		
TT	0 (0.00)	0 (0.00)	–	–	–		
Minor allele T	12 (1.91)	12 (2.17)					
H-W	0.51	0.24					

*H–W* Hardy–Weinberg equilibrium, *P* probability, *OR* odds ratio, *95% CI* 95% confidence interval from two-sided Fisher’s exact test, *χ* ^*2*^_*df = 2*_ *p* Chi-square test for independence with two degrees of freedom for polymorphisms 14 bp ins/del (rs371194629:insATTTGTTCATGCCT/del) in 3′UTR and rs1632947:G>A, *χ* ^*2*^_*df = 4*_ *p* Chi-square test for independence with four degrees of freedom for the polymorphism rs1233334:G>C/T

*Reference

^a^ *P*_corr._ = 0.12

Comparison of patients with minimal and mild (I + II) with moderate and severe (III + IV) endometriosis revealed a protective effect of rs1632947:GG genotype (*P* = 0.04, OR = 0.2, 95% CI = 0.04–0.97), and, in addition, of rs1233334:CT genotype (*P* = 0.04, OR = 0.09, 95% CI = 0.01–0.62; Table 3). These associations lost significance after correction (*P*_corr._ = 0.12 for both comparisons).

**Table 3:** *HLA-G* genotype frequencies in women depending on the severity of endometriosis

Genotype	E I + II (%)	E III + IV (%)	E III + IV vs E I + II						
			*P*	OR	95% CI	Test for independence		Test for trend	
						*p*	χ^2^	*p*	χ^2^
rs371194629:ins/del	*N* = 16	*N* = 187							
Del/del*	8 (50.00)	62 (33.16)		1		0.24	2.89	0.52	0.41
Ins/del	4 (25.00)	87 (46.52)	0.13	2.81	(0.81–9.74)				
Ins/ins	4 (25.00)	38 (20.32)	1.00	1.23	(0.35–4.35)				
Minor allele ins	12 (37.50)	163 (43.58)							
rs1632947:G>A	*N* = 16	*N* = 187							
AA*	2 (12.50)	56 (29.95)		1		**0.05**	**5.86**	**0.022**	**5.26**
AG	6 (37.50)	87 (46.52)	0.71	0.52	(0.10–2.66)				
GG	8 (50.00)	44 (23.53)	**0.04** ^**a**^	**0.20**	**(0.04–0.97)**				
Minor allele A	10 (31.25)	199 (53.21)							
rs1233334:G>C/T	*N* = 16	*N* = 187							
CC*	8 (50.00)	132 (70.59)		1		**0.05**	**9.27**	**0.013**	**6.15**
CG	5 (31.25)	44 (23.53)	0.33	0.53	(0.17–1.72)				
GG	1 (6.25)	5 (2.67)	0.32	0.30	(0.03–2.91)				
GT	0 (0.00)	3 (1.60)	1.00	0.45	(0.02–9.43)				
CT	2 (12.50)	3 (1.60)	**0.04** ^**b**^	**0.09**	**(0.01–0.62)**				
TT	0 (0.00)	0 (0.00)							
Minor allele T	2 (6.25)	6 (1.60)							

*E I + II* endometriosis I + II, *E III + IV* endometriosis III + IV, *P* probability, *OR* odds ratio, *95% CI* 95% confidence interval from two-sided Fisher’s exact test, *χ* ^*2*^_*df = 2*_ *p* Chi-square test for independence with two degrees of freedom for the 14 bp ins/del (rs371194629:insATTTGTTCATGCCT/del) in 3′UTR and rs1632947:G>A polymorphisms, *χ* ^*2*^_*df = 4*_ *p* Chi-square test for independence with four degrees of freedom for the polymorphism rs1233334:G>C/T, *χ* ^*2*^_*df = 1*_ *p* Chi-square test for trend with one degree of freedom for all tested polymorphisms; *Reference

^a^ *P*_corr._ = 0.12

^b^ *P*_corr._ = 0.12

Analysis of peritoneal vs ovarian localization of lesions showed protective effects of rs1632947:GG genotype against ovarian endometriosis (*P* = 0.028, *P*_corr._ = 0.08, OR = 0.16, 95% CI = 0.03–0.84), whereas rs1233334:CT genotype gave a significant result only for peritoneal vs ovarian plus peritoneal endometriosis (*P* = 0.01, *P*_corr._ = 0.03, OR = 0.02, 95% CI = 0.001–0.53). Analysis of all rs1233334 genotypes revealed even higher significance (*p* = 0.006, χ^2^ = 14.35; Table 4). On the other hand, no association with any form of endometriosis was found for the 14 base pair insertion/deletion polymorphism (rs371194629) in the *HLA-G* gene (Tables 2, 3, 4 and Table S2).

**Table 4:** *HLA-G* genotype frequencies in women from endometriosis groups depending on the localization of lesions

Genotype	Endometriosis peritoneal only	Endometriosis ovarian only	Endometriosis ovarian + peritoneal	Endometriosis peritoneal only vs endometriosis ovarian only					Endometriosis peritoneal only vs endometriosis ovarian + peritoneal					Endometriosis ovarian only vs endometriosis ovarian + peritoneal				
				*P*	OR	95% CI	Test for independence		*P*	OR	95% CI	Test for independence		*P*	OR	95% CI	Test for independence	
							*p*	χ^2^				*p*	χ^2^				*p*	χ^2^
rs371194629:ins/del	*N* = 16	*N* = 136	*N* = 92															
Del/del*	8 (50.00)	43 (31.62)	34 (36.95)		1		0.23	2.97		1		0.38	1.94		1		0.70	0.72
Ins/del	4 (25.00)	30 (22.06)	18 (19.57)	0.76	1.40	(0.38–5.06)			1.00	1.06	(0.28-4.00)			0.57	0.76	(0.36–1.59)		
Ins/ins	4 (25.00)	63 (46.32)	40 (43.48)	0.12	2.93	(0.83–10.35)			0.22	2.35	(0.65–8.50)			0.54	0.80	(0.44–1.46)		
Minor allele ins	12 (37.50)	156 (57.35)	98 (53.26)															
rs1632947:G>A	*N* = 16	*N* = 136	*N* = 92															
AA*	2 (12.50)	44 (32.35)	26 (28.26)		1		**0.042**	**6.33**		1		0.28	2.57		1		0.29	2.49
AG	7 (43.75)	67 (49.26)	41 (44.57)	0.48	0.44	(0.90–2.19)			0.47	0.45	(0.09–2.34)			1.00	1.04	(0.56–1.93)		
GG	7 (43.75)	25 (18.38)	25 (27.17)	**0.028** ^**a**^	**0.16**	**(0.03–0.84)**			0.15	0.27	(0.05–1.45)			0.19	1.69	(0.81–3.54)		
Minor allele A	11 (34.38)	155 (56.99)	129 (47.43)															
rs1233334:G>C/T	*N* = 16	*N* = 136	*N* = 92															
CC*	7 (43.75)	101 (74.26)	64 (69.57)		1		0.07	8.80		1		**0.006**	**14.35**		1		0.29	5.02
CG	6 (37.50)	28 (20.59)	23 (25.00)	0.08	0.32	(0.10–1.04)			0.19	0.42	(0.13–1.38)			0.51	1.30	(0.69–2.45)		
GG	1 (6.25)	2 (1.47)	3 (3.26)	0.20	0.14	(0.01–1.72)			0.37	0.33	(0.03–3.60)			0.38	2.37	(0.38–14.56)		
GT	0 (0.00)	1 (0.74)	2 (2.17)	1.00	0.22	(0.01–5.93)			1.00	0.58	(0.03–13.30)			0.56	3.16	(0.28–35.54)		
CT	2 (12.50)	4 (2.94)	0 (0.00)	0.07	0.14	(0.02–0.89)			**0.01** ^**b**^	**0.02**	**(0.001–0.53)**			0.30	0.17	(0.01–3.30)		
TT	0 (0.00)	0 (0.00)	0 (0.00)	–	–	–			–	–	–			–	–	–		
Minor allele T	2 (6.25)	5 (1.84)	2 (1.09)															

*P* probability, *OR* odds ratio, *95% CI* 95% confidence interval from two-sided Fisher’s exact test, *χ* ^*2*^_*df = 2*_ *p* Chi-square test for independence with two degrees of freedom for the 14 bp ins/del (rs371194629:ins ATTTGTTCATGCCT/del) in 3′UTR and rs1632947:G>A polymorphisms, *χ* ^*2*^_*df = 4*_ *p* Chi-square test for independence with four degrees of freedom for the polymorphism of the rs1233334:G>C/T

*Reference

^a^ *P*_corr._ = 0.08

^b^ *P*_corr._ = 0.03

### *LILRB1* and *LILRB2* but not *KIR2DL4* polymorphisms are associated with endometriosis

*LILRB1* rs41308748:G>A polymorphism was distributed differently between patients and controls (*P* = 0.0048, *P*_corr._ = 0.024, OR = 4.62, 95% CI = 1.52–14.02 for AA genotype, and *p* = 0.0035, *χ* ^2^ = 11.33 for all genotypes; Table 5). Similar results were found by analysis according to the stage of the disease (*P* = 0.007, *P*_corr._ = 0.035, OR = 4.8, 95% CI = 1.52–15.15 for AA, and *p* = 0.007, *χ* ^2^ = 9.93 for all genotypes), and localization of lesions (*P* = 0.011, *P*_corr._ = 0.055, OR = 4.24, 95% CI = 1.36–13.21 for AA and *p* = 0.01, *χ* ^2^ = 9.12 for all genotypes; Table S3). The frequency of other examined SNPs did not differ between analyzed groups (Table S3).

**Table 5:** *LILRB1, LILRB2* and *KIR2DL4* genotype and minor allele frequencies in women from Control and Endometriosis groups

Genotype	Control (%)	Patients (%)	Patients vs Control				
			*P*	OR	95% CI	Test for independence	
						*p*	χ^2^
LILRB1 rs41308748:G>A	*N* = 314	*N* = 272					
GG*	261 (83.12)	226 (83.09)		1		**0.0035**	**11.33**
GA	49 (15.61)	30 (11.03)	0.18	0.71	(0.43–1.15)		
AA	4 (1.27)	16 (5.88)	**0.0048** ^**a**^	**4.62**	**(1.52–14.02)**		
Minor allele A	57 (9.08)	62 (11.40)					
H-W	0.33	0.00					
LILRB1 rs1061680:T>C	*N* = 314	*N* = 272					
TT*	191 (60.82)	176 (64.71)		1		0.14	3.88
TC	112 (35.67)	80 (29.41)	0.18	0.76	(0.54–1.10)		
CC	11 (3.51)	16 (5.88)	0.32	1.58	(0.71–3.50)		
Minor allele C	134 (21.34)	112 (20.59)					
H-W	0.27	0.097					
LILRB2 rs383369:G>A	*N* = 314	*N* = 272					
AA*	226 (71.97)	186 (68.38)		1		0.23	2.91
AG	82 (26.12)	84 (30.88)	0.27	1.25	(0.87–1.79)		
GG	6 (1.91)	2 (0.74)	0.31	0.41	(0.08–2.03)		
Minor allele G	94 (14.97)	88 (16.18)					
H-W	0.65	0.022					
LILRB2 rs7247538:C>T	*N* = 314	*N* = 272					
TT*	107 (34.08)	94 (34.56)		1		0.58	1.10
CT	146 (46.50)	134 (49.26)	0.85	1.05	(0.73–1.50)		
CC	61 (19.42)	44 (16.18)	0.47	0.82	(0.51–1.32)		
Minor allele C	268 (42.68)	222 (40.81)					
H-W	0.38	0.74					
KIR2DL4 rs649216:T>C	*N* = 314	*N* = 276					
TT*	103 (32.80)	93 (33.70)		1		0.26	2.70
CT	150 (47.77)	116 (42.03)	0.45	0.86	(0.59–1.24)		
CC	61 (19.43)	67 (24.28)	0.42	1.22	(0.78–1.90)		
Minor allele C	272 (43.31)	250 (45.29)					
H-W	0.63	0.012					

*H–W* Hardy–Weinberg equilibrium, *P* probability, *OR* odds ratio, *95% CI* 95% confidence interval from two-sided Fisher’s exact test, *χ* ^*2*^_*df = 2*_ *p* Chi-square test for independence with two degrees of freedom for all tested polymorphisms; For four samples from the endometriosis group we could not perform *LILRB1* and *LILRB2* genotyping because of a lack of DNA

*Reference

^a^ *P*_corr._ = 0.024

*LILRB2 *rs383369:AG genotype was almost five times more frequent in severe stages (III + IV) of endometriosis than in milder (I + II) stages (*P* = 0.043, *P*_corr._ = 0.215, OR = 7.02, 95% CI = 0.90–54.43, Table 6). A similar, albeit no significant difference was seen in comparison of peritoneal only with peritoneal + ovarian endometriosis (*P* = 0.09, OR = 3.8, 95% CI = 0.81–17.77; Table S4).

**Table 6:** Comparison of the *LILRB* and *KIR2DL4* polymorphisms in women depending on the severity of endometriosis

Genotype	E I + II (%)	E III + IV (%)	E III + IV vs E I + II						
			*P*	OR	95% CI	Test for independence		Test for trend	
						*p*	χ^2^	*p*	χ^2^
LILRB1 rs41308748:G>A	*N* = 16	*N* = 183							
GG*	13 (81.25)	150 (81.97)		1		0.43	1.70	0.68	0.17
GA	3 (18.75)	21 (11.48)	0.44	0.61	(0.16–2.31)				
AA	0 (0.00)	12 (6.55)	0.60	2.24	(0.13–40.02)				
Minor allele A	3 (9.38)	45 (12.30)							
LILRB1 rs1061680:T>C	*N* = 16	*N* = 183							
TT*	12 (75.00)	122 (66.67)		1		0.63	0.93	0.38	0.76
TC	4 (25.00)	53 (28.96)	0.78	1.30	(0.40–4.23)				
CC	0 (0.00)	8 (4.37)	1.00	1.74	(0.09–31.90)				
Minor allele C	4 (12.5)	69 (18.85)							
LILRB2 rs383369:G>A	*N* = 16	*N* = 183							
AA*	15 (93.75)	124 (67.76)		1		0.06	5.49	**0.024**	**5.10**
AG	1 (6.25)	58 (31.69)	**0.043** ^**a**^	**7.02**	**(0.90–54.43)**				
GG	0 (0.00)	1 (0.55)	1.00	0.37	(0.01–9.58)				
Minor allele G	1 (3.13)	60 (16.39)							
LILRB2 rs7247538:C>T	*N* = 16	*N* = 183							
TT*	6 (37.50)	64 (34.97)		1		0.51	1.34	0.69	0.16
CT	6 (37.50)	91 (49.73)	0.56	1.42	(0.44–4.61)				
CC	4 (25.00)	28 (15.30)	0.72	0.66	(0.17–2.51)				
Minor allele C	14 (43.75)	147 (40.16)							
KIR2DL4 rs649216:T>C	*N* = 16	*N* = 187							
TT*	6 (37.50)	60 (32.09)		1		0.23	2.95	0.62	0.25
CT	4 (25.00)	85 (45.45)	0.33	2.13	(0.57–7.86)				
CC	6 (37.50)	42 (22.46)	0.56	0.70	(0.21–2.32)				
Minor allele C	16 (50.00)	169 (45.19)							

*P* probability, *OR* odds ratio, *95% CI* 95% confidence interval from two-sided Fisher’s exact test, *χ* ^*2*^_*df = 2*_ *p* Chi-square test for independence with two degrees of freedom for all tested polymorphisms, *χ* ^*2*^_*df = 1*_ *p* Chi-square test for trend with one degree of freedom for all tested polymorphisms; *Reference; for four samples from the III + IV endometriosis group we could not perform *LILRB1* and *LILRB2* genotyping because of a lack of DNA

^a^ *P*_corr._ = 0.215

Neither the other *LILRB2* SNP (rs7247538:T>C) nor *KIR2DL4* (rs649216:T>C) or *LILRB1 *(rs41308748:G>A and rs1061680:T>C) was distributed differently between mild and severe disease (Table 6). None of other polymorphisms was associated with localization of lesions (Table S4).

## Discussion

In the present study we found that susceptibility to and the severity of endometriosis are associated with polymorphisms in the *HLA-G, LILRB1* and *LILRB2* genes. On the other hand, the disease was not associated with the *KIR2DL4* polymorphism. The data on HLA-G expression in endometrial tissue from healthy individuals and patients with endometriosis are controversial. HLA-G has been detected on eutopic endometrial cells and peritoneal fluid cells in the menstrual phase of women with or without endometriosis (Kawashima et al. 2009); however, Barrier et al. (2006) found HLA-G protein and mRNA expression only in ectopic endometrial tissue but not in eutopic endometrium in women with or without endometriosis, independently of cycle stage. Notably, in an earlier study, Hornung et al. (2001) did not detect HLA-G in peritoneal fluid, ectopic and normal endometrial tissues and stromal cells from endometriosis patients or controls.

The HLA-G molecule exists as seven protein isoforms as a result of alternative splicing: four membrane-bound (HLA-G1, G2, G3, G4) and three soluble (HLA-G5, G6, G7) isoforms (Menier et al. 2010; Donadi et al. 2011; Castelli et al. 2014). Soluble HLA-G (sHLA-G) was found in the peritoneal fluid in similar concentrations in control subjects and in mild and severe endometriosis (Eidukaite and Tamosiunas 2008).

Several important regulatory motifs have been described in the promoter of the *HLA-G* gene, e.g. Enhancer-A (EnhA), the interferon-stimulated response element (ISRE) and the SXY module. All of them are mainly responsible for controlling gene expression by affecting transcription factor binding or promoter methylation (Donadi et al. 2011; Castelli et al. 2014; Persson et al. 2017; Verloes et al. 2017). These regions exhibit many polymorphic sites; among them, positions − 964, − 725 and − 716 (in the promoter) may affect expression of HLA-G (Donadi et al. 2011; Castelli et al. 2014; Persson et al. 2017; Verloes et al. 2017; Amodio et al. 2016; Ober et al. 2003). Indeed, we found here protective effects of rs1632947:GG (− 964GG) and rs1233334:CT (− 725CT) *HLA-G* genotypes on susceptibility to endometriosis and/or progression of the disease (Table 7). On the other hand, a 14 bp insertion/deletion in the 3′UTR (rs371194629) has an influence on both expression and alternative splicing of HLA-G (Verloes et al. 2017) and the level of sHLA-G (Chen et al. 2008). However, no association of this polymorphism with endometriosis was seen in our study. The reason why one polymorphism, rs1632947:GG genotype in the promoter region, increasing expression of HLA-G (Ober et al. 2006), seems to protect against endometriosis, whereas 14 bp deletion in 3′UTR (rs371194629), also increasing HLA-G expression (Verloes et al. 2017), had no effect, needs explanation by further experiments. No other reports on the role of *HLA-G* polymorphisms in endometriosis have been published so far. However,it is worth to mention that other class of MHC genes located near HLA-G (HLA-DQ and HLA-DRB1) have already been published in the context of endometriosis (Zong et al. 2002; Sundqvist et al. 2011; Sobalska-Kwapis et al. 2017).

**Table 7:** Summarized effect of *HLA-G* and *LILRB* polymorphisms on susceptibility to and severity of endometriosis

Polymorphism	Associated genotype	Comparison	Table	Effect
HLA-G rs1632947:G>A	GG GG GG	Patients vs control Patients according to the rAFS vs control Patients according to the localization of lesions vs control	2 S2 S2	↓ ↓ ↓
HLA-G rs1632947:G>A	GG	Severity III + IV vs I + II	3	↓
HLA-G rs1233334:G>C/T	CT	Severity III + IV vs I + II	3	↓
HLA-G rs1632947:G>A	GG	Ovarian only vs peritoneal only	4	↓
HLA-G rs1233334:G>C/T	CT	Ovarian + peritoneal vs peritoneal only	4	↓
	CT	Ovarian only vs peritoneal only	4	↓?
LILRB1 rs41308748:G>A	AA AA AA	Patients vs control Patients according to the rAFS vs control Patients according to the localization of lesions vs Control	5 S3 S3	↑ ↑ ↑
LILRB2 rs383369:G>A	AG AG	Severity III + IV vs I + II Peritoneal only vs ovarian + peritoneal	6 S4	↑ ↑?

*↓* protection, *↑* susceptibility

The putative role of HLA-G in the etiopathogenesis of endometriosis may be strengthened by our further observation that the disease is also associated with polymorphism in *LILRB1* and *LILRB2* genes coding for HLA-G receptors. NK cells express different levels of LILRB1 (Kirwan and Burshtyn 2005) and individuals vary in its positivity, ranging from 10 to 77% of NK cells, depending on gene polymorphism (Davidson et al. 2010).

The rs41308748:G>A polymorphism of the *LILRB1* gene is an intronic SNP situated between the cytoplasmic tail and the 3′UTR sequence, which could have an influence on the splicing process. We found its association (AA genotype) with susceptibility to endometriosis (Table 7); therefore, studies on splicing variants in endometriosis would be desirable. We observed earlier a protective effect of the GA genotype in recurrent miscarriage, whereas the AA genotype had no effect (Nowak et al. 2016). The rs1061680:T>C is a non-synonymous SNP, located in the sequence encoding the extracellular D2 domain (Davidson et al. 2010). It is in strong linkage disequilibrium with another SNP (rs10423364:A>G) which is located in a potential transcription factor binding site (our in silico analysis) and may therefore affect gene expression. Thus, rs1061680:T>C may be a marker of rs10423364:A>G, and may also influence protein structure. However, in our present study we did not reveal its association with endometriosis.

The polymorphism rs7247538:C>T of *LILRB2* changes histidine to tyrosine (p. His300Tyr) in the amino acid sequence of the protein. Our in silico analysis indicated that it may also have a possibly damaging influence on the splicing process. However, this polymorphism was not associated with endometriosis. The second tested SNP in the *LILRB2* gene was the rs383369:G>A (p. Arg20His) and it has been located in the signal sequence region. The G allele of rs383369 has been associated with low expression levels of LILRB2 in Northeast Asians, where it has a high frequency; however, it is infrequent in Europeans (Hirayasu et al. 2008). In our population, almost all individuals possessed the alternative A allele, and GG homozygotes were virtually absent. Nevertheless, the AG heterozygotes had 7 times higher probability of having severe endometriosis than AA homozygotes (Table 6). It suggests, then, that lower LILRB2 expression may predispose to more severe stages of the disease.

KIR2DL4 has been considered to be also an HLA-G receptor (Rajagopalan and Long 2012, 2014). Its long cytoplasmic tail suggests an inhibitory function. However, it has only one immunoreceptor tyrosine inhibitory motif (ITIM) in the cytoplasmic tail and a positively charged arginine residue in its transmembrane region, allowing it to complex with the FcεRI-*γ* chain which transduces the activation signal upon ligand binding by KIR2DL4 (Kikuchi-Maki et al. 2005). However, the HLA-G/KIR2DL4 interaction has recently been questioned (Le Page et al. 2014). In addition, only one out of four individuals in our population possesses a functional receptor (Nowak et al. 2015b). The lack of functional KIR2DL4 may be compensated by the presence of LILRB1. Notably, LILRB1, despite its inhibitory potential, may also exert an activating effect through its immunoreceptor tyrosine-based switch motif (ITSM) (Li et al. 2009) and therefore substitute for KIR2DL4.

There are some limitations of our work. First, the group of subjects with minimal or mild endometriosis was small (16 individuals). This resulted from late diagnosis, as women often do not see their doctor until they suffer from infertility or the pain becomes unbearable. Second, protein expression of cell surface LILRB1, LILRB2 and KIR2DL4 as well as soluble or membrane HLA-G was not examined here. However, this will be a future direction of our research, with particular emphasis on expression of these molecules in endometriotic lesions in peritoneum vs ovary. Moreover, recently published GWAS analysis of potential protein-modifying genetic variants in 9000 endometriosis patients and 150,000 controls of European ancestry (Sapkota et al. 2017b) have not identified our proposed variants with endometriosis pathogenesis. However, variants which modify protein structure through amino acid substitutions or alter stop signals or splicing, particularly those with MAF < 0.05 have been implicated as important but not well covered in GWA studies. Moreover, only about 18% of endometriosis cases in Sapkota et al. (2017b) samples had moderate-to-severe disease while in our study these stages accounted to 92%, and therefore Sapkota et al. (2017b) analysis may not have adequate reference in severe cases. In addition, the cost of whole genome or exome sequencing methods limits large-scale studies and it still limits the selection of potential SNPs for testing.

In conclusion, our results suggest that HLA-G and its receptors LILRB1 and LILRB2, but not KIR2DL4, may play a role in elimination of ectopic endometrial cells and in development of the disease. Our data are novel, as this is the first report on this topic.

## Electronic supplementary material

Below is the link to the electronic supplementary material.

**Table S1** PCR conditions for *LILRB1* (rs41308748:G>A) and *LILRB2* (rs383369:G>A, rs7247538:C>T) genotyping (DOCX 13 KB)

**Table S2** *HLA-G* genotype and minor allele frequencies in women from Control and Endometriosis groups (DOC 65 KB)

**Table S3** *LILRB1, LILRB2* and *KIR2DL4* genotype and minor allele frequencies in women from Control and Endometriosis groups (DOC 75 KB)

**Table S4** Comparison of the *LILRB* and *KIR2DL4* polymorphisms in women depending on the localization of lesions (DOC 85 KB)

**Fig S1** Scatter plot of representative results from the real-time PCR of HLA-G rs1233334:G>C/T SNP genotyping (TIF 244 KB)
