# Supplementary material for: The impact of HLA-G, LILRB1 and LILRB2 gene polymorphisms on susceptibility to and severity of endometriosis
Source: Mol Genet Genomics. 2017 Dec 12;293(3):601–13. doi: 10.1007/s00438-017-1404-3 (PMC5948266; doi:10.1007/s00438-017-1404-3)
Supplement: Supplementary file 1 — Table S1 PCR conditions for LILRB1 (rs41308748:G>A) and LILRB2 (rs383369:G>A, rs7247538:C>T) genotyping (DOCX 13 KB) [file 438_2017_1404_MOESM1_ESM.docx]

**The impact of *HLA-G*, *LILRB1* and *LILRB2* gene polymorphisms on susceptibility to and severity of endometriosis**

**Molecular Genetics and Genomics**

Aleksandra Bylińska, Karolina Wilczyńska, Jacek Malejczyk, Łukasz Milewski, Marta Wagner, Monika Jasek, Wanda Niepiekło-Miniewska, Andrzej Wiśniewski, Rafał Płoski, Ewa Barcz, Piotr Roszkowski, Paweł Kamiński, Andrzej Malinowski, Jacek R. Wilczyński_,_ Paweł Radwan, Michał Radwan, Piotr Kuśnierczyk, Izabela Nowak

**Corresponding authors:** Department of Clinical Immunology, Laboratory of Immunogenetics and Tissue Immunology, Hirszfeld Institute of Immunology and Experimental Therapy, Polish Academy of Sciences, ul. Rudolfa Weigla 12, 53-114 Wrocław, Poland; Izabela Nowak: izan@iitd.pan.wroc.pl; Piotr Kuśnierczyk: pkusnier@iitd.pan.wroc.pl

**Table S1** PCR conditions for *LILRB1* (rs41308748) and *LILRB2* (rs383369, rs7247538) genotyping

| SNP | Primer sequences (forward, reverse) | Annealing temperature | Restriction enzyme | Digestion temperature, time |
| --- | --- | --- | --- | --- |
| rs41308748 | CACGTTCCTTCCCTCTCACT,  CTCCCATGCATTCCAGACT | 57ºC | AciI | 37ºC, 3h |
| rs383369 | TCAGGCTTCAGGGGGCAAATC,  ATGCTGATTTTTTCTCCCTATAT | 53ºC | TaiI | 65ºC, 3h |
| rs7247538 | CAAGGAGGGGGAACGTGACCTTCG,  GCCTCATCCTGGCCATCACTAATTGGAT | 66ºC | Hpy166II | 37ºC, 3h |
