# Supplementary material for: The impact of HLA-G, LILRB1 and LILRB2 gene polymorphisms on susceptibility to and severity of endometriosis
Source: Mol Genet Genomics. 2017 Dec 12;293(3):601–13. doi: 10.1007/s00438-017-1404-3 (PMC5948266; doi:10.1007/s00438-017-1404-3)
Supplement: Supplementary file 2 — Table S2 HLA-G genotype and minor allele frequencies in women from Control and Endometriosis groups (DOC 65 KB) [file 438_2017_1404_MOESM2_ESM.doc]

**The impact of *HLA-G*, *LILRB1* and *LILRB2* gene polymorphisms on susceptibility to and severity of endometriosis**

**Molecular Genetics and Genomics**

Aleksandra Bylińska, Karolina Wilczyńska, Jacek Malejczyk, Łukasz Milewski, Marta Wagner, Monika Jasek, Wanda Niepiekło-Miniewska, Andrzej Wiśniewski, Rafał Płoski, Ewa Barcz, Piotr Roszkowski, Paweł Kamiński, Andrzej Malinowski, Jacek R. Wilczyński, Paweł Radwan, Michał Radwan, Piotr Kuśnierczyk, Izabela Nowak

**Corresponding authors:** Department of Clinical Immunology, Laboratory of Immunogenetics and Tissue Immunology, Hirszfeld Institute of Immunology and Experimental Therapy, Polish Academy of Sciences, ul. Rudolfa Weigla 12, 53-114 Wrocław, Poland; Izabela Nowak: izan@iitd.pan.wroc.pl; Piotr Kuśnierczyk: pkusnier@iitd.pan.wroc.pl

**Table S2** *HLA-G* genotype and minor allele frequencies in women from Control and Endometriosis groups

| Genotype | Control (%) | Endometriosis according to the rAFS (%) | Endometriosis according to the localization of lesions (%) | Endometriosis according to the rAFS  vs Control | | | | | Endometriosis according to the localization  of lesions vs Control | | | | |
| --- | --- | --- | --- | --- | --- | --- | --- | --- | --- | --- | --- | --- | --- |
|  |  |  |  | P | OR | 95% CI | Test for independence | | P | OR | 95% CI | Test for independence | |
|  |  |  |  |  |  |  | *p* | χ^2^ |  |  |  | *p* | χ^2^ |
| rs371194629:ins/del | N = 314 | N = 203 | N = 244 |  |  |  |  |  |  |  |  |  |  |
| Del/del* | 113 (35.99) | 70 (34.48) | 85 (34.84) |  | 1 |  | 0.49 | 1.42 |  | 1 |  | 0.35 | 2.10 |
| Ins/del | 149 (47.45) | 91 (44.93) | 107 (43.85) | 1.00 | 0.99 | (0.66-1.47) |  |  | 0.85 | 0.95 | (0.66-1.39) |  |  |
| Ins/ins | 52 (16.56) | 42 (20.69) | 52 (21.31) | 0.30 | 1.29 | (0.78-2.16) |  |  | 0.27 | 1.33 | (0.83-2.14) |  |  |
| Minor allele ins | 253 (40.29) | 175 (43.10) | 211 (43.24) |  |  |  |  |  |  |  |  |  |  |
| rs1632947:G>A | N = 314 | N = 203 | N=244 |  |  |  |  |  |  |  |  |  |  |
| AA* | 63 (20.06) | 58 (28.57) | 72 (29.51) |  | 1 |  | 0.08 | 5.08 |  | 1 |  | **0.024** | **7.49** |
| AG | 157 (50.00) | 93 (45.81) | 115 (47.13) | 0.06 | 0.64 | (0.41-1.00) |  |  | 0.45 | 0.65 | (0.42-0.97) |  |  |
| GG | 94 (29.94) | 52 (25.62) | 57 (23.36) | **0.05^a^** | **0.60** | **(0.37-0.98)** |  |  | **0.009^b^** | **0.53** | **(0.33-0.85)** |  |  |
| Minor allele A | 283 (45.06) | 209 (51.48) | 259 (53.07) |  |  |  |  |  |  |  |  |  |  |
| rs1233334:G>C/T | N = 314 | N = 203 | N=244 |  |  |  |  |  |  |  |  |  |  |
| CC* | 215 (68.47) | 140 (168.97) | 172 (70.49) |  | 1 |  | 0.61 | 2.71 |  | 1 |  | 0.67 | 2.35 |
| CG | 79 (25.16) | 49 (24.14) | 57 (23.36) | 0.83 | 0.95 | (0.63-1.44) |  |  | 0.62 | 0.90 | (0.61-1.34) |  |  |
| GG | 8 (2.55) | 6 (2.96) | 6 (2.46) | 0.79 | 1.15 | (0.39-3.39) |  |  | 1.00 | 0.94 | (0.32-2.75) |  |  |
| GT | 1 (0.32) | 3 (1.48) | 3 (1.23) | 0.31 | 4.61 | (0.47-44.76) |  |  | 0.33 | 3.75 | (0.39-36.39) |  |  |
| CT | 11 (3.50) | 5 (2.46) | 6 (2.46) | 0.61 | 0.70 | (0.24-2.05) |  |  | 0.62 | 0.68 | (0.25-1.88) |  |  |
| TT | 0 (0.00) | 0 (0.00) | 0 (0.00) | - | - | - |  |  | - | - | - |  |  |
| Minor allele T | 12 (1.91) | 8 (1.97) | 9 (1.84) |  |  |  |  |  |  |  |  |  |  |

H-W, Hardy-Weinberg equilibrium; P, probability; OR, odds ratio; 95% CI, 95% confidence interval from two-sided Fisher’s exact test; χ^2^_df=2_, *p* chi-square test for independence with two degree of freedom for polymorphisms 14 bp ins/del (rs371194629:ins ATTTGTTCATGCCT/del) in 3’UTR and rs1632947:G>A; χ^2^_df=4_, *p* chi-square test for independence with four degree of freedom for the polymorphism of the rs1233334:G>C/T; rAFS, revised American Fertility Society; *Reference; ^a^*P_corr._* = 0.15; ^b^*P_corr._* = 0.027
