# Supplementary material for: The impact of HLA-G, LILRB1 and LILRB2 gene polymorphisms on susceptibility to and severity of endometriosis
Source: Mol Genet Genomics. 2017 Dec 12;293(3):601–13. doi: 10.1007/s00438-017-1404-3 (PMC5948266; doi:10.1007/s00438-017-1404-3)
Supplement: Supplementary file 3 — Table S3 LILRB1, LILRB2 and KIR2DL4 genotype and minor allele frequencies in women from Control and Endometriosis groups (DOC 75 KB) [file 438_2017_1404_MOESM3_ESM.doc]

**The impact of *HLA-G*, *LILRB1* and *LILRB2* gene polymorphisms on susceptibility to and severity of endometriosis**

**Molecular Genetics and Genomics**

Aleksandra Bylińska, Karolina Wilczyńska, Jacek Malejczyk, Łukasz Milewski, Marta Wagner, Monika Jasek, Wanda Niepiekło-Miniewska, Andrzej Wiśniewski, Rafał Płoski, Ewa Barcz, Piotr Roszkowski, Paweł Kamiński, Andrzej Malinowski, Jacek R. Wilczyński, Paweł Radwan, Michał Radwan, Piotr Kuśnierczyk, Izabela Nowak

**Corresponding authors:** Department of Clinical Immunology, Laboratory of Immunogenetics and Tissue Immunology, Hirszfeld Institute of Immunology and Experimental Therapy, Polish Academy of Sciences, ul. Rudolfa Weigla 12, 53-114 Wrocław, Poland; Izabela Nowak: izan@iitd.pan.wroc.pl; Piotr Kuśnierczyk: pkusnier@iitd.pan.wroc.pl

**Table S3** *LILRB1*, *LILRB2* and *KIR2DL4* genotype and minor allele frequencies in women from Control and Endometriosis groups

| Genotype | Control (%) | Endometriosis according to the rAFS (%) | Endometriosis according to the localization of lesions (%) | Endometriosis according to the rAFS  vs Control | | | | | Endometriosis according to the localization  of lesions vs Control | | | | |
| --- | --- | --- | --- | --- | --- | --- | --- | --- | --- | --- | --- | --- | --- |
|  |  |  |  | P | OR | 95% CI | Test for independence | | P | OR | 95% CI | Test for independence | |
|  |  |  |  |  |  |  | *p* | χ^2^ |  |  |  | *p* | χ^2^ |
| LILRB1 rs41308748:G>A | N = 314 | N = 199 | N = 241 |  |  |  |  |  |  |  |  |  |  |
| GG* | 261 (83.12) | 163 (81.91) | 200 (82.99) |  | 1 |  | **0.007** | **9.93** |  | 1 |  | **0.01** | **9.12** |
| GA | 49 (15.61) | 24 (12.06) | 28 (11.62) | 0.43 | 0.78 | (0.46-1.33) |  |  | 0.26 | 0.75 | (0.45-1.23) |  |  |
| AA | 4 (1.27) | 12 (6.03) | 13 (5.39) | **0.007^a^** | **4.80** | **(1.52-15.15)** |  |  | **0.011^b^** | **4.24** | **(1.36-13.21)** |  |  |
| Minor allele A | 57 (9.08) | 48 (12.06) | 54 (11.20) |  |  |  |  |  |  |  |  |  |  |
| LILRB1 rs106168:T>C | N = 314 | N = 199 | N = 241 |  |  |  |  |  |  |  |  |  |  |
| TT* | 191 (60.82) | 134 (67.34) | 151 (62.65) |  | 1 |  | 0.26 | 2.73 |  | 1 |  | 0.31 | 2.37 |
| TC | 112 (35.67) | 57 (28.64) | 76 (31.54) | 0.12 | 0.73 | (0.49-1.07) |  |  | 0.41 | 0.86 | (0.60-1.23) |  |  |
| CC | 11 (3.51) | 8 (4.02) | 14 (5.81) | 1.00 | 1.04 | (0.41-2.65) |  |  | 0.30 | 1.61 | (0.71-3.65) |  |  |
| Minor allele C | 134 (21.34) | 73 (18.34) | 104 (21.58) |  |  |  |  |  |  |  |  |  |  |
| LILRB2 rs383369:G>A | N = 314 | N = 199 | N = 241 |  |  |  |  |  |  |  |  |  |  |
| AA* | 226 (71.97) | 139 (69.85) | 164 (68.05) |  | 1 |  | 0.30 | 2.40 |  | 1 |  | 0.13 | 4.13 |
| AG | 82 (26.12) | 59 (29.65) | 76 (31.54) | 0.48 | 1.17 | (0.79-1.74) |  |  | 0.22 | 1.28 | (0.88-1.85) |  |  |
| GG | 6 (1.91) | 1 (0.50) | 1 (0.41) | 0.26 | 0.27 | (0.03-2.28) |  |  | 0.25 | 0.23 | (0.03-1.93) |  |  |
| Minor allele G | 94 (14.97) | 61 (15.33) | 78 (16.18) |  |  |  |  |  |  |  |  |  |  |
| LILRB2 rs724753:C>T | N = 314 | N = 199 | N = 241 |  |  |  |  |  |  |  |  |  |  |
| TT* | 107 (34.08) | 70 (35.18) | 82 (34.02) |  | 1 |  | 0.63 | 0.92 |  | 1 |  | 0.41 | 1.76 |
| CT | 146 (46.50) | 97 (48.74) | 122 (50.62) | 1.00 | 1.02 | (0.68-1.51) |  |  | 0.70 | 1.09 | (0.75-1.59) |  |  |
| CC | 61 (19.42) | 32 (16.08) | 37 (15.35) | 0.43 | 0.80 | (0.48-1.35) |  |  | 0.38 | 0.79 | (0.48-1.31) |  |  |
| Minor allele C | 268 (42.68) | 161 (40.45) | 196 (40.66) |  |  |  |  |  |  |  |  |  |  |
| KIR2DL4 rs649216:T>C | N = 314 | N = 203 | N = 244 |  |  |  |  |  |  |  |  |  |  |
| TT* | 103 (32.80) | 66 (32.51) | 81 (33.20) |  | 1 |  | 0.48 | 1.46 |  | 1 |  | 0.27 | 2.63 |
| CT | 150 (47.77) | 89 (43.84) | 103 (42.21) | 0.76 | 0.93 | (0.62-1.39) |  |  | 0.49 | 0.87 | (0.59-1.28) |  |  |
| CC | 61 (19.43) | 48 (23.65) | 60 (24.59) | 0.45 | 1.23 | (0.75-2.00) |  |  | 0.35 | 1.25 | (0.79-1.98) |  |  |
| Minor allele C | 272 (43.31) | 185 (45.57) | 223 (49.78) |  |  |  |  |  |  |  |  |  |  |

H-W, Hardy-Weinberg equilibrium; P, probability; OR, odds ratio; 95% CI, 95% confidence interval from two-sided Fisher’s exact test; χ^2^_df=2_, *p* chi-square test for independence with two degree of for all tested polymorphisms; rAFS, revised American Fertility Society; *Reference; ^a^*P_corr._* = 0.035; ^b^*P_corr._* = 0.055
