# Supplementary material for: The impact of HLA-G, LILRB1 and LILRB2 gene polymorphisms on susceptibility to and severity of endometriosis
Source: Mol Genet Genomics. 2017 Dec 12;293(3):601–13. doi: 10.1007/s00438-017-1404-3 (PMC5948266; doi:10.1007/s00438-017-1404-3)
Supplement: Supplementary file 4 — Table S4 Comparison of the LILRB and KIR2DL4 polymorphisms in women depending on the localization of lesions (DOC 85 KB) [file 438_2017_1404_MOESM4_ESM.doc]

**The impact of *HLA-G*, *LILRB1* and *LILRB2* gene polymorphisms on susceptibility to and severity of endometriosis**

**Molecular Genetics and Genomics**

Aleksandra Bylińska, Karolina Wilczyńska, Jacek Malejczyk, Łukasz Milewski, Marta Wagner, Monika Jasek, Wanda Niepiekło-Miniewska, Andrzej Wiśniewski, Rafał Płoski, Ewa Barcz, Piotr Roszkowski, Paweł Kamiński, Andrzej Malinowski, Jacek R. Wilczyński, Paweł Radwan, Michał Radwan, Piotr Kuśnierczyk, Izabela Nowak

**Corresponding authors:** Department of Clinical Immunology, Laboratory of Immunogenetics and Tissue Immunology, Hirszfeld Institute of Immunology and Experimental Therapy, Polish Academy of Sciences, ul. Rudolfa Weigla 12, 53-114 Wrocław, Poland; Izabela Nowak: izan@iitd.pan.wroc.pl; Piotr Kuśnierczyk: pkusnier@iitd.pan.wroc.pl

**Table S4** Comparison of the *LILRB* and *KIR2DL4* polymorphisms in women depending on the localization of lesions

| Genotype | Endometriosis peritoneal only | Endometriosis ovarian only | Endometriosis ovarian+peritoneal | Endometriosis peritoneal only vs endometriosis ovarian only | | | | | Endometriosis peritoneal only vs endometriosis ovarian + peritoneal | | | | | Endometriosis ovarian only vs endometriosis ovarian + peritoneal | | | | |
| --- | --- | --- | --- | --- | --- | --- | --- | --- | --- | --- | --- | --- | --- | --- | --- | --- | --- | --- |
|  |  |  |  | P | OR | 95% CI | Test for independence | | P | OR | 95% CI | Test for independence | | P | OR | 95% CI | Test for independence | |
|  |  |  |  |  |  |  | p | χ^2^ |  |  |  | p | χ^2^ |  |  |  | p | χ^2^ |
| LILRB1 rs41308748:G>A | N = 16 | N = 134 | N = 91 |  |  |  |  |  |  |  |  |  |  |  |  |  |  |  |
| GG | 13 (81.25) | 112 (83.58) | 75 (82.42) |  | 1 |  | 0.40 | 1.83 |  | 1 |  | 0.51 | 1.34 |  | 1 |  | 0.92 | 0.16 |
| GA | 3 (18.75) | 14 (10.45) | 11 (12.09) | 0.41 | 0.54 | (0.14-2.14) |  |  | 0.46 | 0.64 | (0.16-2.59) |  |  | 0.83 | 1.17 | (0.51-2.72) |  |  |
| AA | 0 (0.00) | 8 (5.97) | 5 (5.49) | 1.00 | 2.04 | (0.11-37.38) |  |  | 1.00 | 1.97 | (0.10-37.70) |  |  | 1.00 | 0.93 | (0.29-2.96) |  |  |
| Minor allele A | 3 (9.38) | 30 (11.19) | 21 (11.54) |  |  |  |  |  |  |  |  |  |  |  |  |  |  |  |
| LILRB1 rs1061680:T>C | N = 16 | N = 134 | N = 91 |  |  |  |  |  |  |  |  |  |  |  |  |  |  |  |
| TT | 11 (68.75) | 79 (58.96) | 61 (67.03) |  | 1 |  | 0.72 | 0.66 |  | 1 |  | 0.98 | 0.05 |  | 1 |  | 0.46 | 1.57 |
| TC | 4 (25.00) | 47 (35.07) | 25 (27.47) | 0.57 | 1.64 | (0.49-5.43) |  |  | 1.00 | 1.13 | (0.33-3.88) |  |  | 0.24 | 0.69 | (0.38-1.24) |  |  |
| CC | 1 (6.25) | 8 (5.97) | 5 (31.25) | 1.00 | 1.11 | (0.13-9.79) |  |  | 1.00 | 0.90 | (0.1-8.48) |  |  | 0.78 | 0.81 | (0.25-2.60) |  |  |
| Minor allele C | 6 (18.75) | 63 (23.51) | 35 (19.23) |  |  |  |  |  |  |  |  |  |  |  |  |  |  |  |
| LILRB2 rs383369:G>A | N = 16 | N = 134 | N = 91 |  |  |  |  |  |  |  |  |  |  |  |  |  |  |  |
| AA | 14 (87.5) | 91 (67.91) | 59 (64.84) |  | 1 |  | 0.27 | 2.63 |  | 1 |  | 0.07 | 3.22 |  | 1 |  | 0.61 | 0.99 |
| AG | 2 (12.5) | 42 (31.34) | 32 (35.16) | 0.15 | 3.23 | (0.70-14.87) |  |  | 0.09 | 3.80 | (0.81-17.77) |  |  | 0.66 | 1.18 | (0.67-2.07) |  |  |
| GG | 0 (0.00) | 1 (0.75) | 0 (0.00) | 1.00 | 0.48 | (0.02-12.25) |  |  | - | - | - |  |  | 1.00 | 0.51 | (0.02-12.80) |  |  |
| Minor allele G | 2 (6.25) | 44 (16.42) | 32 (17.58) |  |  |  |  |  |  |  |  |  |  |  |  |  |  |  |
| LILRB2 rs7247538:C>T | N = 16 | N = 134 | N = 91 |  |  |  |  |  |  |  |  |  |  |  |  |  |  |  |
| TT | 5 (31.25) | 47 (35.07) | 30 (32.97) |  | 1 |  | 0.93 | 0.15 |  | 1 |  | 0.90 | 0.21 |  | 1 |  | 0.87 | 0.27 |
| CT | 8 (50.00) | 66 (49.25) | 48 (52.75) | 1.00 | 0.88 | (0.27-2.85) |  |  | 1.00 | 1.00 | (0.30-3.34) |  |  | 0.76 | 1.14 | (0.63-2.06) |  |  |
| CC | 3 (18.75) | 21 (15.67) | 13 (14.29) | 0.70 | 0.74 | (0.16-3.41) |  |  | 0.69 | 0.72 | (0.15-3.48) |  |  | 1.00 | 0.97 | (0.42-2.22) |  |  |
| Minor allele C | 14 (43.75) | 108 (40.30) | 74 (40.66) |  |  |  |  |  |  |  |  |  |  |  |  |  |  |  |
| KIR2DL4 rs649216:T>C | N=16 | N=136 | N=92 |  |  |  |  |  |  |  |  |  |  |  |  |  |  |  |
| TT | 5 (31.25) | 52 (38.24) | 24 (26.09) |  | 1 |  | 0.31 | 2.37 |  | 1 |  | 0.55 | 1.18 |  | 1 |  | 0.13 | 4.05 |
| CT | 5 (31.25) | 56 (41.18) | 42 (45.65) | 1.00 | 1.08 | (0.29-3.94) |  |  | 0.49 | 1.75 | (0.46-6.67) |  |  | 0.16 | 1.63 | (0.87-3.05) |  |  |
| CC | 6 (37.50) | 28 (20.59) | 26 (28.26) | 0.32 | 0.45 | (0.13-1.60) |  |  | 1.00 | 0.90 | (0.24-3.35) |  |  | 0.07 | 2.01 | (0.98-4.14) |  |  |
| Minor allele C | 17 (53.12) | 112 (41.18) | 94 (51.09) |  |  |  |  |  |  |  |  |  |  |  |  |  |  |  |

P, probability; OR, odds ratio; 95% CI, 95% confidence interval from two-sided Fisher’s exact test; χ^2^_df=2_, *p* chi-square test for independence with two degree of for all tested polymorphisms; *Reference
