# Supplementary figures and images for: The impact of HLA-G, LILRB1 and LILRB2 gene polymorphisms on susceptibility to and severity of endometriosis
Source: Mol Genet Genomics. 2017 Dec 12;293(3):601–13. doi: 10.1007/s00438-017-1404-3 (PMC5948266; doi:10.1007/s00438-017-1404-3)

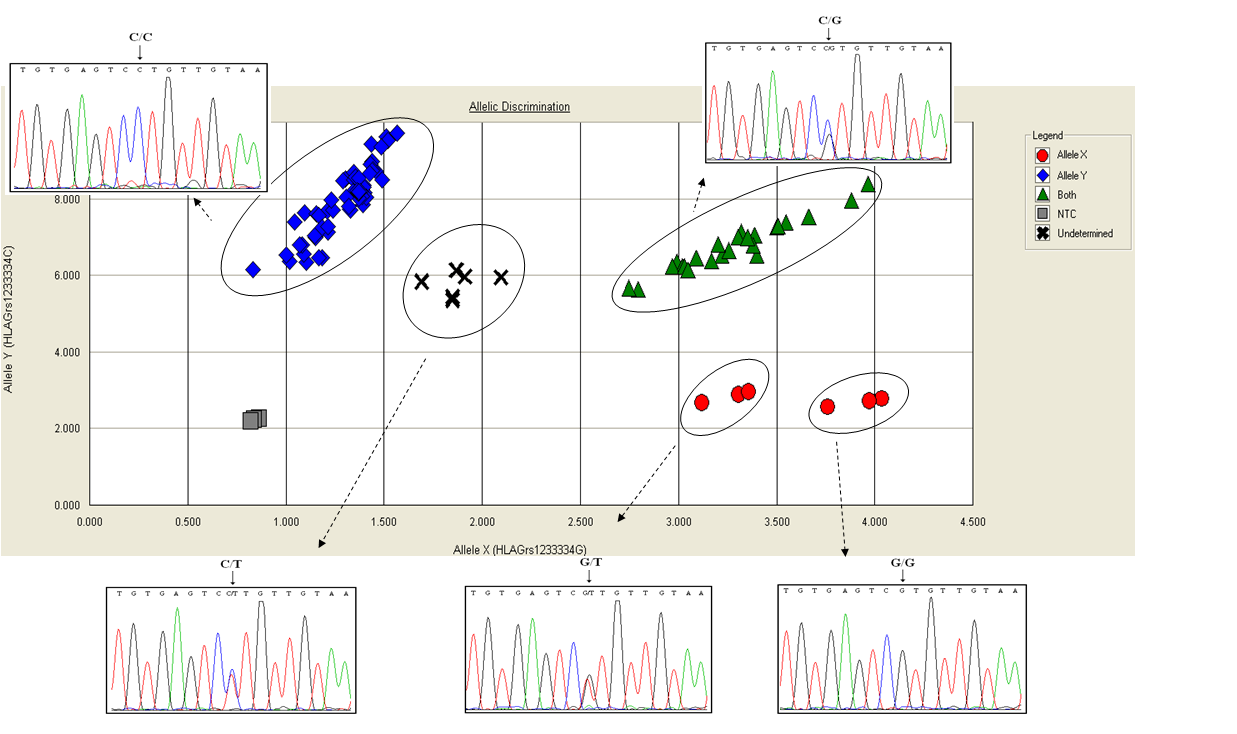

Supplement: Supplementary file 5 — Fig S1 Scatter plot of representative results from the real-time PCR of HLA-G rs1233334:G>C/T SNP genotyping (TIF 244 KB) [file 438_2017_1404_MOESM5_ESM.tif]
